# Family Involvement During Patient Hospitalisation—Developing and Testing a Clinical Decision Aid

**DOI:** 10.1111/scs.70017

**Published:** 2025-03-10

**Authors:** Leah Sejrup Christensen, Mette Hulbæk Andersen, Anette Brink, Eva Hoffmann

**Affiliations:** ^1^ Department of Internal Medicine University Hospital of Southern Denmark Aabenraa Denmark; ^2^ Department of Gynecology and Obstetrics University Hospital of Southern Denmark Aabenraa Denmark; ^3^ Department of Regional Health Research University of Southern Denmark Odense Denmark; ^4^ University College South Denmark Aabenraa Denmark

**Keywords:** acute hospitalisation, chronic disease, qualitative research, shared decision making

## Abstract

**Background:**

The rising prevalence of multi‐morbidity increases treatment complexity and caregiving demands, often necessitating involvement of family members as informal caregivers. While essential, this involvement can be burdensome, causing distress for family members. Shared decision‐making facilitates communication and supports the alignment of patients' and families' preferences and needs with care and treatment decisions. Involving family during patient hospitalisation can be essential as the whole family is affected by illness.

**Aim:**

This project aimed to develop and test a decision aid to systematise family involvement during patient hospitalisation.

**Research Methods:**

The project was based on the theoretical framework of family nursing, and the Danish Patient Decision Aid template guided the process. The decision options, pros and cons were based on 22 patient and 16 family interviews, which were thematically analysed. Six patients, two family members and nine healthcare professionals alpha tested the decision‐aid prototype, which was later beta tested in real‐life clinical settings at five internal medical wards.

**Findings:**

Three themes emerged: (A) *‘*involving family when needed’, (B) *‘*waiting for ward rounds*’* and (C) *‘*involving family with technology’, informing the decision‐aid prototype which consisted of five option cards: (1) ‘I will involve my family myself’, (2) *‘*I do not want to involve my family’, (3) ‘Family wants to be present physically’, (4) ‘Family wants to participate by phone’ and (5) *‘*Family wants to participate by video’. The cards included pros/cons of each option. Alpha testing showed high acceptability and usability, and no alterations were made to the prototype.

**Conclusion:**

The structured patient decision aid enabled a systematic approach to involve the patient's family. It facilitated meaningful conversations between healthcare professionals, patients and family members. The decision aid identified and addressed patients' and family members' specific needs and preferences during hospitalisation.

## 1 Introduction

The concepts of family nursing are perhaps more crucial now than ever before. The incidence of multi‐disease is increasing, likely resulting in more complex treatment and care needs [1, 2, 3, 4]. In 2023, the association of municipalities reported on the incidence of chronic disease in Denmark: one in five people has a chronic disease, and one in seven has two or more chronic diseases [5]. We are experiencing an overall strain on the healthcare system, a lack of healthcare professionals (HCPs) and a focus on early discharge. Therefore, the need for informal caregivers or care coordinators appears increasingly necessary [6, 7].

In Denmark, there is a correlation between the decreased length of hospital stay and a more extensive need for home rehabilitation [8]. This draws further attention to the roles of informal caregivers both during and after hospitalisation. Extensive research has established that family involvement is essential for patients and family members but can be distressing and burdensome [9, 10, 11, 12, 13]. A Danish report draws further attention to family members experiencing the consequences of a lack of resources and organisational shortcomings, highlighting the need for successful collaboration between the patient's family and HCPs in both the primary care setting and the hospitals [14].

Wright and Leahey state, ‘Nurses have an ethical and moral obligation to involve families in their health‐care practice’ [15] as rehabilitation engages HCPs, families and the patient's social network. However, any involvement of patients' families, first and foremost, depends on patients' consent and their right to choose not to involve others [16].

Shared decision‐making (SDM) is a way of communicating with patients, ensuring that patient preferences are the basis for decisions about care, treatment, rehabilitation, etc., and emphasising the right to decline an intervention [17, 18]. Involving family members during hospitalisation can be a vital part of planning discharge and rehabilitation as the whole family is affected by illness [10, 13, 15, 19].

In order to involve family members in a meaningful way during hospitalisation, we must know if the patients want family involvement and gain an overview of the family composition, ranks and boundaries [15]. What are the commitments between family members? Patient decision aids (PtDA) are designed to help facilitate SDM between HCPs and patients, improving the quality of treatment decisions [17]. Expanding that perception to include family members can enable patients to express their preferences for family involvement and for family members to express theirs.

This project was based on the theoretical framework of family nursing and SDM, as SDM may ease the burden on some families while recognising them as a vital part of patient care during hospitalisation [15, 17, 20]. PtDAs are often used to facilitate treatment decisions [21, 22, 23], making their use relevant when framing the question of family involvement during hospitalisation.

### 1.1 Aim

This research project aimed to develop a decision aid to systematise decisions about family involvement during patient hospitalisation, with patients defining family as ‘who they say they are’ [24].

## 2 Methods and Materials

The method was guided by the Danish PtDA template and a generic decision‐helper software developed by the Centre for Shared Decision Making, Lillebaelt Hospital, Denmark [25, 26], meeting the quality criteria of the International Patient Decision Aid Standards (IPDAS) [27]. These SDM tools are based on patient‐centred care, enhancing communication between patient and HCP in several aspects [27, 28]. IPDAS covers 11 core domains: development process of the decision aid, providing balanced information, communicating probabilities of outcomes, clarifying values, using personal stories, guidance and decision coaching, disclosing conflicts of interest, health literacy, basing information on scientific evidence, measuring effectiveness and implementation of PtDAs [29].

Our PtDA was designed to be a part of conversations between patients, family members and nurses/aides within the first couple of days after admission. Acknowledging that HCPs provide patients and family members with opportunities to engage in various inpatient activities, we wanted to promote equal opportunities for deliberation on participation.

Thus, the content of our PtDA prototype was based on patient and family interviews. We employed a hermeneutic approach when collecting and analysing the interview data, acknowledging the intersubjectivity between interviewees and interviewers [30]. All experienced nurses, the interviewers brought their professional background into their interactions with patients and families and the analytic process, acknowledging the interpretive role of the researcher's prior knowledge and experiences in understanding the data. The PtDA prototype was initially alpha‐tested [31, 32, 33], followed by beta testing in a real‐world clinical setting as an add‐on to standard treatment [33].

Three female nurse specialists (the first author and two colleagues) led the project, collected data, managed the PtDA software and facilitated alpha and beta testing. Nurse specialists have insight into clinical practice as caregivers *and* specialists in the clinical field.

**FIGURE 1:**
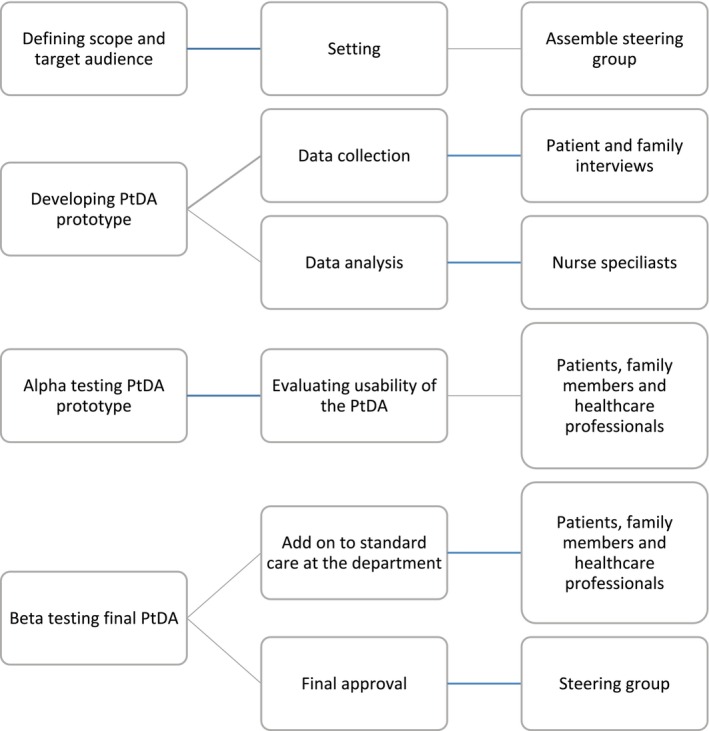
Development process and testing of the patient decision aid for family involvement during hospitalisation.

An overview of the design method is illustrated in Figure 1.

To enhance reporting of this project, we adhere to the IPDAS collaboration template for documenting the development process [33] and the COREQ criteria for reporting qualitative research [34].

### 2.1 Defining Scope and Target Audience

Defining scope includes describing the target audience for our PtDA. The target audience was acutely hospitalised adult patients and adult family members able to take part in a decision. Kicking off the project, we hypothesised that family involvement is ubiquitous during patient hospitalisation, and we need to know who and if, when/how to involve them. A PtDA could assist HCPs, patients and family members in visualising and clarifying expectations to one another during hospitalisation regarding the level and manner of family involvement.

### 2.2 Setting

The project was performed at a public regional hospital in Denmark. The Danish healthcare system offers treatment free of charge, as it is funded through taxes. The hospital involved had approximately 40,000 patient admissions in 2023 [35].

The PtDA was developed at the Department of Internal Medicine, encompassing six specialised wards addressing medical conditions such as kidney disease, diabetes, geriatric syndromes, gastric cancer, lung cancer and acute diseases. During 2023, 9357 patients were admitted to the Department of Internal Medicine, 46.2% being female (7326) and 53.8% male (5031). The mean age of hospitalised patients in the department was 72.6 for females and 72.8 for males.

### 2.3 Assemble Steering Group

A steering group of relevant stakeholders was established. It consisted of two SDM experts from the hospital's quality department, the department chief nurse, six head nurses and six physicians, and three patients from the hospital user‐involvement council. These stakeholders offered a wide range of experience and expertise and were regularly consulted about project progress, findings and testing. The Centre for Shared Decision Making, Lillebaelt Hospital, Denmark, supervised the PtDA development process and guided the use of their decision‐helper software [36].

### 2.4 Developing PtDA Prototype

Developing the PtDA prototype was an iterative process involving designated steering group members and the three nurse specialists. The Danish PtDA design consists of a generic frame slightly altered to the option, option insert cards with pros and cons, and patient/family stories [25, 26]. Patient and family interviews informed the options, pros and cons in our PtDA.

#### 2.4.1 Data Collection (Patient and Family Interviews)

The purpose of data collection was to include ‘typical’ patient/family members from all wards in the department. Inclusion criteria included speaking Danish, being 18 years or older, and being admitted to one of the six wards at the Department of Internal Medicine. We used purposive sampling [37], including patients and family members during hospitalisation and in the daytime. Patients and family members could participate regardless of each other's participation. However, family member participation always preceded the patient's implied acceptance.

Exclusion criteria were patients/family members not cognitively able to carry a conversation. The nurse coordinator on the ward identified participants not considered eligible, and the three nurse specialists approached eligible patients and family members.

The interview focused on patients' and family members' perspectives on family involvement during hospitalisation. Data collection included sex, age and the relationship between patients and family members, which the interviewee reported verbally. Other demographic data were considered outside the project's scope.

We used a semi‐structured interview guide with nine open‐ended questions; for example:

‘What does involvement mean to you?’

‘What is important to you if/when we involve your family during the hospitalisation?’

‘Can you describe how you have been involved as family during the hospitalisation?’

The steering group and experts from the Centre for Shared Decision‐Making developed the interview guide. Interview data consisted of handwritten field notes without voice recordings and included some quotations. All field notes were transcribed into a Word document and stored at a secure drive.

Information power guided the number of participants for inclusion [38]. However, to ensure usability when implementing the PtDA in six different wards, we included interviews with patients and family members from each of the six wards [32].

### 2.5 Data Analysis

Data analysis was guided by the project aim and inspired by Braun and Clarke's (2022) Reflexive Thematic Analysis, together with SDM theory and the theoretical framework of family nursing [15, 24]. This meant that the codes could also be used as pros and cons in the PtDA, and the themes laid the foundation for developing options for family involvement. The nurse specialists analysed and coded the interview data inductively and discussed the findings.

Firstly, they familiarised themselves with the data. Secondly, content was coded and then combined into themes [39]. They used Post‐it notes to visualise this phase, which were scanned and stored in a secure electronic project folder. Finally, the PtDA insert cards were developed deductively, coherent with the findings in the analysis and the PtDA template [25, 26, 39]. Blank option cards with working notes were used creatively as mock‐ups to conceptualise the PtDA prototype.

### 2.6 Alpha Testing PtDA Prototype

After developing the PtDA prototype, HCPs, leaders, hospitalised patients and family members were invited to participate in alpha testing. Family members could participate in the alpha testing regardless of patient participation; however, only with preceding patient consent.

Alpha testing consisted of a questionnaire. The Centre for Shared Decision Making has previously developed and tested this specific questionnaire for alpha testing based on English versions by Bennett et al. and Stacey et al. [31, 32]. The questionnaire consists of eight questions and measures user preparedness for decision‐making, acceptability and usability of the PtDA. However, this questionnaire has primarily been used to evaluate decision aids based on treatment decisions and not decisions about the extent of family involvement and its performance.

We also invited two users to test the PtDA, one with prior experience developing PtDAs and one without.

The steering group reviewed the questionnaire responses and decided whether to alter the PtDA before continuing with beta testing.

### 2.7 Beta Testing Final PtDA

After alpha testing, the PtDA was beta‐tested in a collaboration between the three nurse specialists and nurses/aids in a real‐life clinical setting as an add‐on to standard care [33]. Before this, HCPs had been trained in SDM and introduced to the alpha‐tested PtDA via three‐hour face‐to‐face courses. HCPs, patients and respective family members were included in beta testing by one of the specialists/nurses/aids and informed of the new decision aid, explaining that the PtDA could support decisions about family involvement during patient hospitalisation. HCPs, patients and family members had the opportunity to give feedback regarding usability and the overall experience of the PtDA to the nurse specialists. Feedback from beta testing was given orally and documented by the nurse specialists in a secure electronic project folder the same day, ensuring the content was anonymised. The steering group was presented with feedback and the PtDA before final approval of implementation.

## 3 Ethics Statement

Patients and family members were given information about the project and verbalised consent before participating in interviews and alpha testing as required by Danish ethical standards. The primary ethical consideration behind this project involved not causing undue stress to patients or family members. Data management complied with the European General Data Protection Regulations and Danish law [40, 41]. Data was stored on a secure logged drive; we did not collect data that could be traced back to the patients or family members. The Danish Scientific Ethical Committee did not require formal approval for this project [42], as it was not a biomedical research project. Hospital management approved the project.

## 4 Findings

### 4.1 Patient and Family Interviews

The three nurse specialists conducted individual semi‐structured interviews at bedsides in January 2023. The nurse specialists all had prior experience interviewing. The duration of the interviews varied between 10 and 20 min as some participants answered briefly and others elaborated on their answers. The nurse specialists had no prior relationships with the participants. In total, 40 patients/family members were asked to participate. In total, 22 patients and 16 family members consented and participated. Two patients did not wish to participate in the interviews as they felt poorly. We interviewed participants in pairs: patient and family member or singularly: patient or family member only. The interviews took place in the inpatients' rooms, primarily bedside and single rooms. However, spacious rooms were shared between two and four patients in the emergency admissions ward, separated by large privacy curtains.

Of the 22 participating patients, nine were female, with a mean age of 73. We interviewed 16 family members, of whom 14 were female, with a mean age of 59. Only one patient and one family member were of an ethnic origin other than Danish. Table 1 presents the demographic data and the corresponding age groups for patient and family participants.

**TABLE 1:** Demographic data on interview participants.

**Participating patients**	***n* = 22**
Female	9
Male	13
Age group	
51–60	1
61–70	8
71–80	5
81–90	7
Unknown	1
Ethnicity	
Danish	21
Other	1
**Participating family members**	***n* = 16**
Female	14
Male	2
Age group	
21–30	1
31–40	1
41–50	2
51–60	2
61–70	5
71–80	3
Unknown	2
Ethnicity	
Danish	15
Other	1
Relationship	
Spouse	4
Adult Child	7
Sibling	1
Adult grandchild	2
Friend/Neighbour	2

### 4.2 Thematic Analysis

Three themes were derived from the analysis: (1) involving family when needed, (2) waiting for ward rounds and (3) involving family with technology. Table 2 presents an example of field notes, coding and corresponding themes from the interviews.

**TABLE 2:** An example of the thematic analysis of family involvement during patient hospitalisation and use of a patient decision aid.

Field notes/quotes	Codes	Themes
Involvement is important, but when the patient can involve her husband herself, she does not feel it is necessary for the staff to do it (female patient, 81–90).	Involvement is important Patients involving family themselves	Involving family when needed
The adult children take turn participating in rounds during the admission. They share information afterwards (…) They would like to have the possibility to participate in rounds, but the waiting is long, sometimes 5–6 h (adult female child, 51–60).	Participating in ward rounds Worth the wait	Waiting for ward rounds

The three themes will be presented narratively, supported by specific content from patient and family interviews including information on male/female, patient/family and age group in brackets.

### 4.3 Themes

#### 4.3.1 Involving Family When Needed

This theme describes a continuum from patients who preferred not to involve their families to those who actively embraced family participation and engagement during hospitalisation. Patients themselves assessed the need for family involvement and informed family members about the hospitalisation as they felt appropriate during their admission.

A male patient (aged 61–70) described how hospitalisation could be overwhelming and that he needed to take things one step at a time. Involving family members would not allow him to be at his own pace. Other patients recognised the need for family members to have an active voice during hospitalisation and wanted them to be active in decision‐making. However, others felt that family members could be too involved or were simply superfluous and spoke very clearly of this. This was addressed by one female patient (aged 81–90), who described family involvement as redundant and emphasised that physical treatment was the most important aspect of her admission.

During interviews, some patients gave examples of how their assessment was related to prior hospitalisations and compared previous experiences with their current situation. Several patients did not feel that the current admission was ‘acute enough’ or anything much different from what they had experienced before. Drawing on previous experience, another female patient (aged 61–70) told us about the hours before her admission, when her husband had been the one to call the ambulance, and that she would text her husband when she felt the need to. Other patients had similar stories; often, one family member was involved in the acute hospitalisation, and that person was kept informed by the patient. The patient then evaluated any need for involvement from other family members, such as adult children.

However, patients unfamiliar with hospitalisation were very appreciative of family involvement. Several participants positively described how family members also felt the need to discuss what happened during the hospitalisation and/or participate in ward rounds. One female family member (aged 41–50) described how caring for her father could be challenging. She explained that being involved not only allowed her to better support her father but also fostered a sense of support from the HCPs towards her.

There were many different perceptions of family involvement. Some patients deemed it necessary because they could not remember well and patients could benefit from having an ‘extra set of ears’. Some family members sought out HCPs during hospital visits for more information, some expected that HCPs would call if necessary, and others described a need to participate with perspectives and experiences from home. During interviews, several patients stated that HCPs should simply ask what they preferred.

#### 4.3.2 Waiting for Ward Rounds

Ward rounds were often mentioned during the interviews as an example of involvement during hospitalisation. Family members described ward rounds as something patients and family members could and should be involved in, and valued the possibility of being there when the patient and physician discussed treatment options.

One female family member (aged 41–50) explained that she had been allowed to listen and ask questions but felt that the HCPs did not address her directly. Rounds were often described as something offering family members an opportunity to ask questions and discuss matters with physicians. Furthermore, ward rounds were an event that could bring the family together—the entire family could discuss what was happening. Involving several family members allowed one family to take turns attending rounds. A female family member (aged 51–60) explained that this arrangement also enabled the family to provide a high level of support to her father during hospitalisation.

Many patients and family members described the high degree of waiting involved in participating in ward rounds and, unfortunately, experiences of waiting to no avail. One daughter expressed concern about waiting due to the strain on her father. She and other family members had waited for the physician by her father's bedside for several hours. At some wards, HCPs tried to coordinate rounds with patients and family members. However, this was not always successful. A male patient (aged 91–90) described how his daughter had inquired about a specific time, but the attending physician had been an hour early. Patients often mentioned that coordinating rounds between family members and HCPs would be beneficial. Few family members described it as challenging to know what to expect during ward rounds. Families were in doubt about participating in rounds, how to gain information, or how to become further involved.

#### 4.3.3 Involving Family With Technology

Many patients did not involve family members during ward rounds if they lived far away or had work, as they did not perceive it as possible. HCPs experience frequent telephone calls in the afternoons from patients' long‐distance family members asking about the outcome of the ward rounds. Patients and family members also described feeling reassured that HCPs would contact families as needed, with the decision to make phone calls based on the patient's physical well‐being. During interviews with patients and families together, it became evident that these expectations were often unspoken, with no verbal agreement among family members.

More family members than patients mentioned video as a possibility to be involved or get information during hospitalisation. However, one male patient (aged 61–70) described how he would FaceTime his wife every day *after* rounds. He had been transferred from another hospital to this, his local hospital and now found it much easier to involve his wife. When asked how he would like his family to be involved, he again mentioned the use of technology.

Some family members had the perception that they would have been more involved if they had been more physically present at the hospital.

### 4.4 PtDA Prototype on Family Involvement

The three themes that emerged from data analysis were used to develop the PtDA prototype, options, pros and cons. Adhering to the criteria for decision aids, one option card should give the patient the possibility to choose not to involve family members. The theme ‘Involving family when needed’ resulted in the option ‘I will involve my family myself’. The themes ‘Waiting for ward rounds’ and ‘Involving family with technology’ revolve around when and how to involve family members. Not wanting to isolate family involvement to ward rounds, but rather invite family members to participate in meals, physiotherapy, dietary consultations, etc., we developed three options ‘Family wants to be present physically, ‘Family wants to participate by phone’, and ‘Family wants to participate by video’. Each card summarised the pros and cons of choosing the particular option. Table 3 below shows each option and the corresponding pros and cons derived from the patient and family interviews.

**TABLE 3:** The five options and the corresponding pros and cons in the PtDA on family involvement during patient hospitalisation [43].

Options	Pros	Cons
I will involve my family myself	You decide what gets said You decide when there is contact Less medicalisation	Speaking about illness can be difficult You alone are responsible for sharing information Your family cannot ask questions to the healthcare professionals themselves
I do not want to involve my family	You only need to take responsibility for yourself and your illness You feel that you are protecting your family by not involving them Less medicalisation	You might end up facing your illness journey alone There are no extra ears available to listen, for example, during ward rounds
Family wants to be present physically	My family member has the opportunity to aid me It gives me comfort when my family can be with me Experiencing a closer relationship Possibility for multiple family members to participate	There can be a lot of waiting My family needs to take time off work Family spends time on transportation
Family wants to participate by phone	Contact can happen anywhere No transportation Less time‐consuming Greater flexibility and less planning	You cannot see who you are speaking with My family is unable to aid me Less close relationship
Family wants to participate by video	You can see who you are talking to Contact can happen anywhere Less time‐consuming No transportation Possibility for multiple family members to participate	It may require IT skills My family is unable to aid me Requires a private place to sit, for example, at the workplace Risk of technical issues Requires planning

PtDA option card number two was considered equivalent to ‘no treatment’ as the patient chose not to involve family members during hospitalisation. Furthermore, the generic frame of the decision aid underscores the fact that patients and/or family members can change their decision just by saying so.

The pros and cons listed on each card were based on interview findings and common sense. For example, the disadvantage of participating by telephone and not seeing who is speaking is common sense. The decision‐helper software offers a wide range of visual illustrations for aids, and new ones can be developed as needed. The patient/family stories card, which is a part of the PtDA, was developed with anonymous quotations from interviews. The PtDA can be accessed on the website of the Centre for Shared Decision Making in Danish [43].

### 4.5 Alpha Testing the PtDA

Before alpha testing, the steering group discussed the optimal timing for introducing the PtDA to patients and family members. During the acute hospitalisation phase, patients undergo stabilisation and diagnostic procedures, making it a sensitive time to allocate resources to the PtDA. Consequently, the steering group decided to exclude the emergency ward from the project's scope moving forward. The emergency admissions ward quickly determines whether patients are transferred to peripheral wards or discharged within 24 h. Thus, management prioritised alpha and beta testing at the five peripheral wards offering intermediate patient care.

Six patients, two family members and nine HCPs alpha‐tested the prototype at two wards in April 2023. We used an adaptive questionnaire. The HCPs expressed difficulty answering some of the questions, for example, regarding the patient‐HCP relationship and the time spent during the consultation, as these were not literal consultations but conversations during hospitalisation.

All patients and family members stated that the PtDA helped prepare them for decisions regarding family involvement. Nearly all patients and family members considered the purpose and overall usability of the decision aid to be clear and easy to understand. Only one participant needed additional information and all patients and family members felt the decision aid would be helpful in conversations with HCPs, particularly during initial hospitalisation.

HCPs participating in alpha testing assessed the usability positively, ranging from ‘a little’ to ‘greatly’ helpful. Others stated that assessing the usability of the PtDA was problematic, as it did not revolve around a treatment option but rather the less tangible concept of family involvement.

Ultimately, no changes to the decision aid were made after feedback from alpha testing.

### 4.6 Beta Testing the PtDA

Beta testing of the PtDA took place between June and December 2023. The nurse specialists used the PtDA with patients, family members and HCPs, rotating across five wards and accumulating over 200 h of testing.

Our findings suggest that patients and family members found the decision aid helpful in clarifying their needs and preferences for family involvement. They welcomed the PtDA, spoke of showing it to other family members or taking it home and discussing their options. It became clear that many patients and family members were unaware that participating in ward rounds online was possible and welcomed the idea.

HCPs expressed concern that the PtDA made conversation feel forced and unnatural. HCPs lacked spontaneity when using the PtDA. This observation, noted by the nurse specialists, may be attributed to their limited experience with the use of PtDAs. The design of the decision aid, the wording and the illustrations were accepted, but usability concerning documentation practice was challenging. They experienced double documentation in the electronic patient records and a lack of overview when recalling decisions made by patients and family members. The HCPs described how they often documented notes on patient–family relationships during ward admission using their standard record activity. HCPs were asked to document family involvement in a designated activity, which resulted in HCPs documenting it twice. Implementing changes to address issues in the electronic records is challenging. No changes to the PtDA were made after beta testing.

## 5 Discussion

This article outlines the development of a PtDA aimed at systematising the option of family involvement during hospitalisation. Our PtDA is not treatment‐based but has the potential to be applied to adult patient trajectories within the healthcare sector, as it was developed within the context of chronic disease and acute hospitalisation. Our PtDA options explore patient preferences and, if granted permission, family preferences.

According to the law, family involvement depends on patient preferences; therefore, patients function as gatekeepers when involving family members in treatment and care [16]. This corresponds with checking if the patient has an emergency contact person listed in the electronic patient record. However, that emergency contact may often be more than that. This person is involved with the patient at some level and cares about what happens at the hospital.

In this project, almost every patient wanted a family member involved. However, the patients seemed to assess when and how much subjectively. Some patients did not disclose their choices and preferences with family, leaving some family members wanting a higher level of involvement than the patients themselves seemed to deem necessary. It is not possible for us to know if this was intended or if the patients simply had not made an active choice. Family members sometimes anticipated that HCPs would provide them with the information they needed. As a result, family expectations did not always align well with patients' actual preferences; this can be described as a false consensus between individuals who assume they share the same perspectives [44].

An acute hospitalisation can increase the risk of false consensus between patient and family, as the patient is subjected to many choices during hospitalisation [44, 45]. When a patient and a family member have not aligned their expectations, for example, on family involvement, it can lead to significant misunderstandings and miscommunication within the healthcare context. Families who appear misaligned may reflect a family system overwhelmed by the hospital experience. According to family systems theory, one of the concepts is that *‘*the family is able to create a balance between change and stability*’* [15]. This concept introduces the perspective of how a family reorganises themselves into a new stability when illness occurs—until it changes again. HCPs are essential in supporting this change as they acknowledge the family is a unit and invite family members to share their perspectives [15]. One way of doing so is through SDM. Patients and family members can be empowered to express individual preferences and needs for involvement when HCPs encourage and facilitate SDM [17, 18, 46].

One essential care task for family members is offering support and understanding the healthcare trajectory [14]; therefore, participating in ward rounds is viewed by patients and family members as essential [46]. Family members often negotiate on behalf of the patient and support them in navigating the healthcare system [17, 47, 48, 49]. The PtDA did not only facilitate a conversation between the family as a whole and the HCPs, but it also supported shared decisions between the patient *and* the family members, enabling both parties to express their preferences. Through this dialogue, important dynamics such as family composition, rank and subsystems may emerge. Another Danish study has explored these family dynamics during acute admission, developing a model conceptualising family involvement through the negotiation power [47]. Hoffmann et al. found that family members were mandated to negotiate and be involved by the patient. Similarly, our PtDA clarifies the level of family involvement and how families can participate. Our findings suggest that ward rounds are pivotal for family members involved during patient hospitalisation, providing them with vital updates on the patient's condition, treatment progress and discharge plans.

The findings of this project suggest that the PtDA may mitigate the risk of overburdening families or excluding them from involvement in a family member's hospitalisation. To what extent do family members wish to participate during hospitalisation? Our PtDA enabled family members to choose between different options, including the option to decline involvement. The five steps in the PtDA, along with authentic patient and family stories, support family members in choosing their preferred level of involvement.

Given the strain on homecare services, there is a growing reliance on family members as informal caregivers [14, 50]. Hospitalisation presents an opportunity to assess family systems and prepare family members for specific responsibilities. The PtDA can help HCPs uncover family obligations and responsibilities post‐discharge, also potentially identifying family members who may feel overwhelmed [15]. There is a risk of overburdening informal caregivers, which becomes particularly problematic as the duration of illness or diminished capacity increases [51]. This risk is higher if the caregiver is female or the relationship can be described as an adult‐child relationship [51]. The family members participating in this project were predominantly female and adult children, highlighting the need for social support such as that provided by SDM and the PtDA. The challenges for informal caregivers will likely increase, as long‐term and chronic diseases increase, urging the healthcare sector to take action.

## 6 Strengths and Limitations

Applying a hermeneutic approach to collecting and analysing data improved our understanding of patient and family needs for involvement during hospitalisation. Surprisingly, only one patient participant wanted no family involvement.

Despite not having the opportunity to observe family involvement longitudinally, credibility was enhanced by interviewing patients and family members at different wards and ensuring nurse specialists did not interview individuals they had cared for. The nurse specialists' dual roles and authors' prior clinical experience were acknowledged during analysis, although formal data triangulation was not conducted.

The development process ensured high quality and reliability by utilising the decision helper software [26] and adhering to the International Patient Decision Aid Standards [29]. Engagement with HCPs, patients and family members provided diverse perspectives that shaped the PtDA content. The iterative approach, including alpha and beta testing, validated the PtDA's usability and effectiveness, while collaboration with the Center for Shared Decision Making and expert consultants further enhanced confirmability. During beta testing, the Danish National Survey of Patient Experience reported increased family involvement during ward rounds and improved family communication for acutely admitted patients in the Department of Internal Medicine [52].

Unlike many existing decision aids tailored to specific diseases or medical scenarios, our PtDA can be used across a diverse group of patients in internal medicine. By focusing on the family unit, the PtDA promotes family nursing, care and planning during patient hospitalisation.

Limitations of this project included that the Danish framework for developing PtDAs was designed for evidence‐based medicine and treatment decisions. We utilised the software from a user perspective, thus not staying within this intended frame. However, the PtDA was developed in collaboration with the founders of the template.

The aim of designing a decision aid for patients *and* family members may be too complex. HCPs noted this complexity, but not patients or family members. The HCPs were novices in SDM, and simply gaining more experience could help overcome barriers towards the PtDA.

While efforts were made to include a wide range of participants, most family members were female. If experiences differ between genders, this could introduce information bias. The generalisability of the PtDA could be improved if there were a stricter focus on maximising variation of sex, age, and family relationships of the participants. Transferability is only supported by information on previous admissions at the department. We did not include detailed descriptions of patient diagnoses and chronic diseases.

Further, a notable limitation is that the patient and family interviews relied solely on handwritten notes without voice recordings. This may have hindered the capture of nuanced verbal expressions and non‐verbal cues, potentially leading to incomplete documentation of participant responses and interpretations. Furthermore, the alpha testing questionnaire was developed for treatment‐based PtDAs, as this PtDA was not, the HCPs did not always find the questions easy to understand in this particular context, and some left out answers. A project log was kept to track meetings, decisions and coordinate tasks.

Acknowledging project limitations, the systematic development adhered to known standards. Project trustworthiness is enhanced through credibility and transparent procedures.

## 7 Conclusion

This project developed an aid to enhance patient decisions about family involvement, supporting patients in clarifying needs and preferences during decision‐making. Using the structure of patient decision aids, we developed a tool that enabled a systematic approach to family involvement during patient hospitalisation. Three themes were derived from the analysis of interview data: (1) involving family when needed, (2) waiting for ward rounds and (3) involving family with technology. The PtDA was based on the experiences of acute and often chronically ill patients and their family, patients who subjectively assessed the need for family involvement without always aligning expectations with family members. We found that family members often viewed ward rounds as pivotal during hospitalisation and that they needed information about the hospitalisation.

HCPs reported that the PtDA was a facilitator for important conversations between patients and families. The PtDA identifies and addresses patients' and families' specific needs and preferences, thereby enabling meaningful involvement during hospitalisation.

## 8 Implications for Practice

HCPs and policymakers must acknowledge that acute disease affects the entire family. Determining family structure and facilitating the preferred level of family involvement during patient hospitalisation is a part of that acknowledgement. Our PtDA can enhance awareness of patients' families; who they are, where they live, what resources the family has and how/when they can be involved. The PtDA can be introduced at any time during hospitalisation if HCPs have knowledge of SDM. Introducing a PtDA facilitating family involvement during hospitalisation could be one of the gateways when exploring the framework of family nursing. Including elements of family systems theory, we could share knowledge across care sectors, and family members might feel more equipped to co‐manage chronic diseases.

## Author Contributions

A.B. had the original project idea. L.S.C. undertook data management, significant parts of data collection, and analysis. L.S.C. and A.B. led the development of the PtDA. L.S.C. tested the PtDA and drafted the initial manuscript. M.H.A. and E.H. guided and gave critical inputs to the manuscript throughout the process.

## Ethics Statement

Ethical review and approval were waived for this study, as Danish law does not require approval or registration of qualitative research or interviews. The head of the department approved the project. Data management complies with the European General Data Protection Regulations and Danish law, and the project complies with national scientific ethical standards.

## Consent

Informed consent was obtained from all subjects involved in the study.

## Conflicts of Interest

The authors declare no conflicts of interest.

## Data Availability

The data that support the findings of this study are available on request from the corresponding author. The data are not publicly available due to privacy or ethical restrictions.
